# The Origin of the Act of 1890

**Published:** 1918-08-31

**Authors:** 


### PAPERS ON MADNESS.

### III.—The Origin of the Act of 1890.

In spite of the obscurantism of the lawyers, as represented by those two shining legal luminaries, Lord Eldon and Lord Brougham, Acts of limited scope, gingerly touching the edges of the matter, were passed from time to time, but nothing of real importance was done until Lord Somerset took the matter in hand in 1842, ably and strenuously assisted by that champion of the weak and the voiceless, Lord Ashley, subsequently the Good Lord Shaftesbury, who was for so many years the chairman of the Lunacy Commission, which he did so much to reconstitute. Lord Somerset's Bill passed into an Act in 1842, and the Commissioners constituted by it at once opened an investigation into the state and treatment of the insane throughout the country. The investigation lasted two years, and the Report of the Commissioners was presented to Parliament in 1844, disclosing a state of things that, although a great and general improvement on what had prevailed earlier in the century, was still very bad. In Dr. Warburton's asylum at Bethnal Green, for instance, the abuses in which were the direct cause of the Commission of Inquiry in 1827, "whereas in 1828 there were commonly 150 to 200 of the patients restrained by leg-locks, chains, and other fetters—certainly during the night—in 1844 there were, out of the 582 patients, only five whose violence rendered this species of restriction necessary, and even the confinement or coercion resorted to was of the most moderate description, and in the opinion of the visiting officers most necessary."

### The Magna Charta of the Mad.

The consequence of this report was the introduction by Lord Ashley of two Bills, one of which established a permanent Commission of Lunacy with extended powers, and the other rendered compulsory the provision of public asylums by counties and boroughs, a provision that up to that time had been only permissive, and had been adopted by but few public authorities. These Bills received the Royal Assent in August 1845, and constitute the celebrated Acts 8 and 9 Vict. c. 100 and c. 106. They constitute what has been well called the Magna Charta of the mad.

### Evading the Asylum.

Amending and consolidating Acts were passed at frequent intervals after this, but no material or fundamental change was made in the law until 1890, when the great Act 53 and 54 Vict. cap. 5 was passed and introduced several important changes in the law. The origin of this Act is interesting as an illustration of the illogical vagaries of the public mind and of the way in which legislation is brought about. It happened that in 1878 the friends of a certain Mrs. Weldon had, or thought they had, reason to suppose that she was not sane, and they consulted a certain Dr. Winslow, who appears to have arrived at the conclusion on their *ex parte* statement that the lady was insane. He was the proprietor or manager of a private asylum, and was willing to receive the lady as a certified patient. Accordingly, he sent two doctors, Dr. Semple and Dr. Rutherford, to her house to examine and, if necessary, to certify her as a lunatic. They did so, and certified her, on grounds that were subsequently found by juries to be insufficient, to be insane. On receiving these certificates, Dr. Winslow sent nurses to Mrs. Weldon's house to fetch her to his asylum, but Mrs. Weldon was forewarned and forearmed. She sent for the police to protect her. She barricaded herself into her own room. She escaped from the house and took refuge with friends, and altogether eluded Dr. Winslow's efforts to capture her; and then the fun began.

### The Lady and the Law.

Mrs. Weldon, whether mad or not, was an extremely clever woman, and Dr. Winslow was an extremely stupid man. The contest was a very unequal one, and Dr. Winslow and the other doctors concerned never had a chance. She brought actions for damages against each of them, and conducted her case herself. She was not only a very clever woman and soon acquired a knowledge of law and procedure sufficient to conduct her own case with propriety, but she proved a most persuasive speaker and a most efficient cross-examiner. Add to this that she was a very handsome woman with a fine presence and a great gift of distinct and deliberate utterance, and what chance had a mere man against her before a British jury, already deeply prejudiced against lunatic asylums by the traditional abuses of the old madhouses? Mrs. Weldon won all along the line. She gained £500 each in damages from the two certifying doctors, and then went for Dr. Winslow as the arch-conspirator and instigator of the whole plot. Here, however, she met with a check. She was non-suited by Baron Huddleston (the last of the Barons), and Dr. Winslow congratulated himself upon his impunity. But his rejoicings were premature. Nothing daunted, Mrs. Weldon took her case to the Court of Appeal, and won it. A new trial was ordered and held, and Dr. Winslow was mulcted in damages to the tune of £1,000. The damages had been laid at £10,000, but Mrs. Weldon was well satisfied, and Dr. Winslow was completely ruined.

### A Gilbertian Situation.

So far, so good. Mrs. Weldon won, as we have said, all along the line. The doctors were incautious, they were satisfied by very insufficient evidence, but there was no reason to doubt the

☛ Previous articles appeared on March 2, and August 24, page 447.

PAPERS ON MADNESS—(*continued*).

*bona fides* of Dr. Semple or Dr. Rutherford. Mrs. Weldon was not, in fact, detained or placed under restraint for a single hour. She sued the certifying doctors for libel, but the libel was contained in the medical certificate that she was of unsound mind, a confidential document that, until published by herself, could never come under the eye of anyone but a few officials. She sued for an assault, which consisted merely in placing a hand upon her shoulder. Her suits were successful, and she completely ruined her antagonists. It would be difficult to contrive or to imagine a more completely successful vindication of the law as it then existed. It showed itself competent to protect Mrs. Weldon, and to inflict condign, if not summary, punishment upon those who had presumed to assert that she was mad and to try to treat her as mad. Yet, so strangely constituted is the human mind, that no sooner had Mrs. Weldon won her verdicts, vindicated her own sanity, and ruined her antagonists, than a furious clamour arose for the alteration and amendment of that law that had proved so efficacious for her protection. Leaders were published in the newspapers, their correspondence columns overflowed with demands for fresh legislation, public meetings were held, and Mr. Bernard Shaw made one of the first of his successful attempts at notoriety by asserting that the authorities of asylums sent out press-gangs to seize sane people and incarcerate them for life. Such a hullabaloo was raised that the Government was compelled to act. It acted slowly, after its manner, and it was twelve years after the attempt on Mrs. Weldon that the Act of 1890, which was due solely to the agitation originated by her, passed through the Houses of Parliament and received the sanction of the Crown.

Periodical Certification and Examination.

The chief novelties in the Lunacy Act, 1890, were two. First, that the two medical certificates hitherto legalising the taking of a lunatic and his detention in an asylum should no longer be sufficient. It was provided that thenceforth these certificates should be placed before a magistrate, and that his order, made upon the evidence of the certificates and such other evidence as he cared to call for, should in future be necessary before an insane person can be placed under care. The second innovation provided for the periodical review of every case of a person detained under care. At the end of one year after admission, and again at the end of two years, and again at the end of four years, seven years, twelve years, and thereafter every five years, the patient is to be examined and certified afresh; and if he cannot then be re-certified, or if the examination is omitted, even accidentally, the patient becomes, *ipso facto*, a free man, and cannot legally be further detained without fresh medical certificates and a fresh magistrate's order, from the date of which the periodical examinations begin again *de novo*.

The latter of these two provisions is undoubtedly of value to the lunatic. In large asylums it prevents his being overlooked and lost in the crowd. It ensures that from time to time special attention shall be paid to him, and his fitness or unfitness for discharge reviewed and determined anew; and thus it is an additional safeguard against undue detention; but it sometimes operates in another way, which may be desirable from the point of view of public policy, but bears hardly on the person it is intended to protect. There is a certain small residue of patients who have lived for many years in a lunatic asylum, who have, after a long residence therein, recovered their wits; who have no relatives, no friends, no connections outside the asylum; who look upon it as their house, and their only home; who are too old to work, and too fixed in their habits to alter them without great discomfort; and who look with consternation and dread upon the prospect of their discharge. True, they might be kept in workhouses for a few pence a week less than it costs to keep them in asylums; but the saving would be so small as to be neglectable; and to turn them forcibly out of the asylum that has become to them a comfortable and happy home, borders upon cruelty. The provision is prompted by humanity and consideration for the feelings and the welfare of the lunatic, but, like so many well-meant provisions of legislation, its effect is in some cases exactly the reverse of that which is designed.

A Disaster to Patient and Friends.

The other new provision of the Lunacy Act, 1890, is less innocuous. It often opposes a real and pernicious obstacle in the way of placing a lunatic under care with the promptitude that is desirable. A magistrate is not always to be had at a moment's notice; and although many magistrates have a considerable experience of lunacy and are thoroughly competent to decide whether or no it is right and expedient to send a patient to an asylum, a few are still imbued with the traditional opinion that the lunatic asylum is a "charnel-house," and that every person alleged to be a lunatic is the victim of a foul plot on the part of his relatives and his doctors for some sordid and nefarious purpose. When the lunatic is, as many lunatics are, a plausible and clever man, excessively mischievous, but certifiable with difficulty, the intervention of a magistrate of this type is a disaster both to the patient and to his friends.

